# High-pulse-energy multiphoton imaging of neurons and oligodendrocytes in deep murine brain with a fiber laser

**DOI:** 10.1038/s41598-021-86924-6

**Published:** 2021-04-12

**Authors:** Michael J. Redlich, Brad Prall, Edesly Canto-Said, Yevgeniy Busarov, Lilit Shirinyan-Tuka, Arafat Meah, Hyungsik Lim

**Affiliations:** 1 Department of Physics and Astronomy, Hunter College, New York, NY 10065 USA; 2 Department of Physics, The Graduate Center of the City University of New York, New York, NY 10016 USA; 3 Clark-MXR, Inc., 7300 W. Huron River Drive, Dexter, MI 48130 USA

**Keywords:** Biomedical engineering, Imaging techniques, Cellular neuroscience

## Abstract

Here we demonstrate high-pulse-energy multiphoton microscopy (MPM) for intravital imaging of neurons and oligodendrocytes in the murine brain. Pulses with an order of magnitude higher energy (~ 10 nJ) were employed from a ytterbium doped fiber laser source at a 1-MHz repetition rate, as compared to the standard 80-MHz Ti:Sapphire laser. Intravital imaging was performed on mice expressing common fluorescent proteins, including green (GFP) and yellow fluorescent proteins (YFP), and TagRFPt. One fifth of the average power could be used for superior depths of MPM imaging, as compared to the Ti:Sapphire laser: A depth of ~ 860 µm was obtained by imaging the Thy1-YFP brain in vivo with 6.5 mW, and cortical myelin as deep as 400 µm ex vivo by intrinsic third-harmonic generation using 50 mW. The substantially higher pulse energy enables novel regimes of photophysics to be exploited for microscopic imaging. The limitation from higher order phototoxicity is also discussed.

## Introduction

Two-photon excitation microscopy (2PM) with descanned detection has a superior depth of imaging, far exceeding that of one-photon excitation microscopy^1–3^. Similar gain can be attained in general for multiphoton microscopy (MPM), expanding biological investigations by light microscopy from cultured cells to fresh thick tissues. By virtue of the safety of the near-infrared (NIR) excitation^4^, MPM has been the primary tool for intravital microscopy leading to breakthrough discoveries in broad biomedical fields. Particularly in neurobiology, 2PM imaging of the mouse brain in vivo has been crucial for studying the structure and function of the neural network. Improving the depth of MPM imaging will be tremendously impactful in other areas as well, such as oncology and immunology^5–7^.

The depth range of MPM depends on the peak power, or the pulse energy, of the excitation. Although highly versatile in terms of the repetition rate and the pulse duration^8–11^, the most common version of mode-locked femtosecond Ti:Sapphire laser for MPM produces output pulses of ~ 100-fs duration with a ~ 100-MHz repetition rate and ~ 1 W of average power, corresponding to a pulse energy of ~ 10 nJ. Then, < 1 nJ is delivered to the live mouse brain to visualize as deep as 500 µm by two-photon excited fluorescence (2PEF). The pulse energy from a light source can be increased further, while maintaining the average power, by lowering the repetition rate. Conversely, a lower repetition rate allows the average power to be reduced without compromising the pulse energy. A 0.2-MHz Ti:Sapphire regenerative amplifier has been employed to deliver ~ 225-nJ pulses for an imaging depth of ~ 1 mm in the mouse brain by 2PEF^12^ and 1-MHz fiber-based light sources have been also demonstrated for MPM^13,14^. A fiber laser is advantageous as a low-repetition-rate light source due to the easy extension of the cavity length, the freedom from free-space alignments, and smaller footprints^15^. However, despite many benefits, the use for intravital MPM imaging has been anecdotal and not yet permeated biomedical research as widely as Ti:Sapphire lasers. One of the major reasons hampering the adoption is that the high pulse energy has not been exploited appropriately, i.e., the average power has been excessive for the achieved depth or the depth of imaging has been inadequate for the average power, raising a question about the pulse quality. Also, the limited tunability is a significant shortcoming.

Here we demonstrate high-pulse-energy MPM for visualizing neurons and oligodendrocytes in live murine brains. A ytterbium (Yb) doped fiber light source (YFLS) was employed, which delivered a pulse energy of ~ 10 nJ at the sample at a repetition rate of 1 MHz. The performance was compared with a 80-MHz Ti:Sapphire laser. The peak power of the YFLS was confirmed by means of second-harmonic generation (SHG) imaging of the retina. Then it was tested for intravital 2PEF imaging of transgenic mice expressing green (GFP) and yellow fluorescent proteins (YFP). Also, a red fluorescent protein (RFP) was examined for multi-color YFLS imaging. Phototoxicity arising from high pulse energy was characterized, as a factor limiting the imaging depth of high-pulse-energy MPM. A strategy of mitigating superficial photodamages was demonstrated for deeper imaging of cortical myelin by third-harmonic generation (THG).

## Results

### Instrument for high-pulse-energy MPM

**Figure 1:**
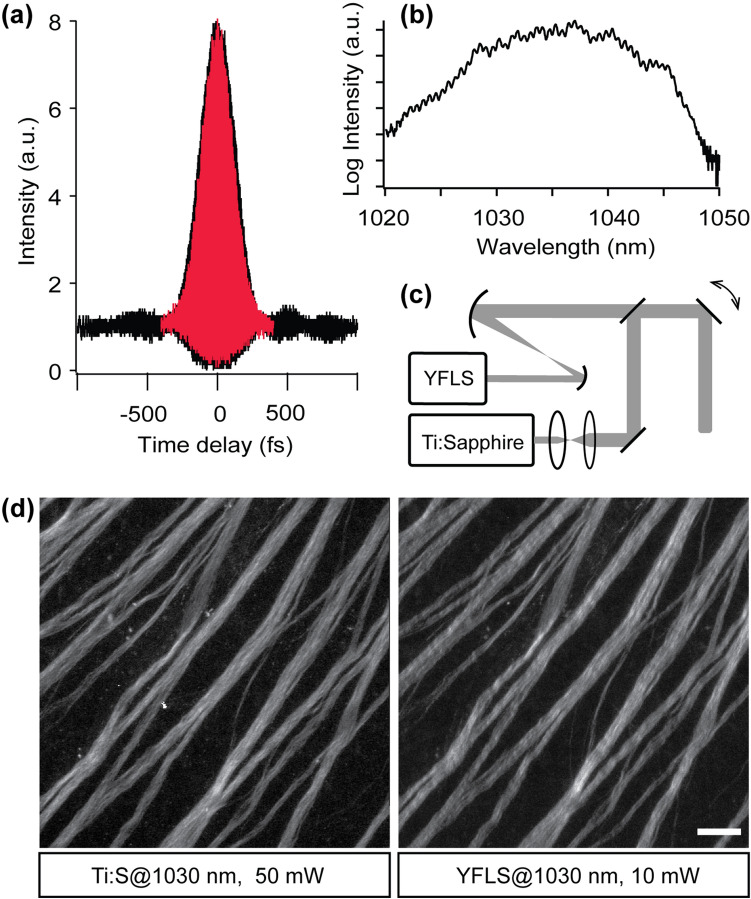
Setup for high-pulse-energy MPM. (**a**) Autocorrelation and (**b**) spectrum of a pulse from the YFLS. Overlaid in red, a pulse from the Ti:Sapphire laser. (**c**) The excitation beam path. (**d**) SHG images of the retinal nerve fiber bundles, confirming the 2PE equivalent average power. Scale bar, 30 µm.

The YFLS was constructed at Clark-MXR for this study. The pulses from a 25-MHz Yb-doped fiber oscillator^16^ were reduced to a repetition rate of 1 MHz by a pulse picker and subsequently amplified to an average power of ~ 0.5 W through a Yb-doped fiber. The output pulse duration and spectral quality were achieved as designed (Fig. 1a,b). The parameters of the YFLS and Ti:Sapphire laser are compared in Table 1.

**Table 1:** The parameters of laser output.

	Ti:Sapphire laser	Yb-doped fiber light source
Center wavelength	700–1050 nm (tunable)	1030 nm (fixed)
Average power	1–3 W	0.5 W
Repetition rate	80 MHz	1 MHz
Pulse duration^a^	160 fs	190 fs

^a^Full width at half maximum (FWHM), assuming a Gaussian pulse.

The high energy of the YFLS pulses required a special consideration for the instrumentation of MPM which was otherwise identical to a standard setup. Since the peak power of the YFLS is ~10 times higher than that of the Ti:Sapphire laser, the corresponding nonlinear length for the YFLS is one hundredth^17^. As a result, an excessive nonlinear phase can accumulate in the propagation through glasses degrading the pulse quality. To reduce the effect, the output beam from the YFLS was attenuated immediately and magnified by a pair of concave spherical mirrors with negligible astigmatism^18^ (Fig. 1c).

### Determination of the equivalent average powers

We compared the peak power of the pulses from the 1-MHz YFLS and the 80-MHz Ti:Sapphire laser. The n-photon excitation (nPE) signal acquired from a molecule per unit time is

1 $$S(z)\approx {\eta }_{n}{\Phi }_{0}exp\left[-\frac{z}{{l}_{\Phi }}\right]{\left[\frac{P}{f\tau }exp\left[-\frac{z}{{l}_{s}}\right]\right]}^{n}f\tau$$

where n is the order of the nPE process, ${\eta }_{n}$ is the quantum efficiency of the nPE process, ${\Phi }_{0}$ is the collection efficiency at the surface, ${l}_{\Phi }$ and ${l}_{s}$ are the characteristic lengths at the emission and excitation wavelengths respectively, $P$ is the average power, $f$ is the repetition rate, and $\tau$ is the pulse duration. The equation, which is a generalization of the previous works^12,19^, can be expressed in a modular form

2 $$S(z)\approx {\Phi }_{0}{\eta }_{n}\cdot \frac{{P}^{n}}{{\left(f\tau \right)}^{n-1}}\cdot exp\left[-\frac{z}{{l}_{n}}\right]={S}_{0}exp\left[-\frac{z}{{l}_{n}}\right]$$

where ${S}_{0}$ is the nPE signal at the surface and the decay length ${l}_{n}$ is

3 $${l}_{n}^{-1}={\left({l}_{s}/n\right)}^{-1}+{l}_{\Phi }^{-1}$$

The average powers of two lasers with different repetition rates and pulse durations are equivalent for an nPE process, i.e., producing the same nPE signal, provided the *equivalent power* is equal.

4 $${P}_{E}=\frac{P}{{\left(f\tau \right)}^{1-\frac{1}{n}}}$$

Evidently, the merit of a low repetition rate is absent for 1PE and increases with the order of the process. The maximum depth of imaging, at which the signal $S({z}_{max})$ is equal to the background $B$, is

5 $${z}_{max}=n\cdot {l}_{n}\cdot \mathit{ln}\left[{\left(\frac{{\eta }_{n}{\Phi }_{0}}{B}\right)}^\frac{1}{n}{P}_{E}\right]$$

To compare the equivalent powers of the 1-MHz YFLS and the 80-MHz Ti:Sapphire laser, we employed SHG signal arising from uniformly polarized microtubules in the retinal nerve fiber bundles^20^. A fresh flatmounted retina from a C57BL/6 mouse was imaged by the two excitation sources tuned to the same wavelength of 1030 nm. The average power of the Ti:Sapphire laser beam was continuously adjusted until the SHG signal was identical to that of the YFLS. The best match was found at an average power of 10 and 50 mW, respectively, indicating a fold change in ${\left(f\tau \right)}^\frac{1}{2}$ of 5 (Fig. 1d). It was a bit lower than the predicted value of 8.2 from the parameters of Table 1, which could be due to the non-Gaussian temporal shape of the YFLS pulse (Fig. 1a). Consequently, it was estimated that the ratio of the 3PE equivalent power should be 8.6.

### Imaging genetically encoded fluorophores

**Figure 2:**
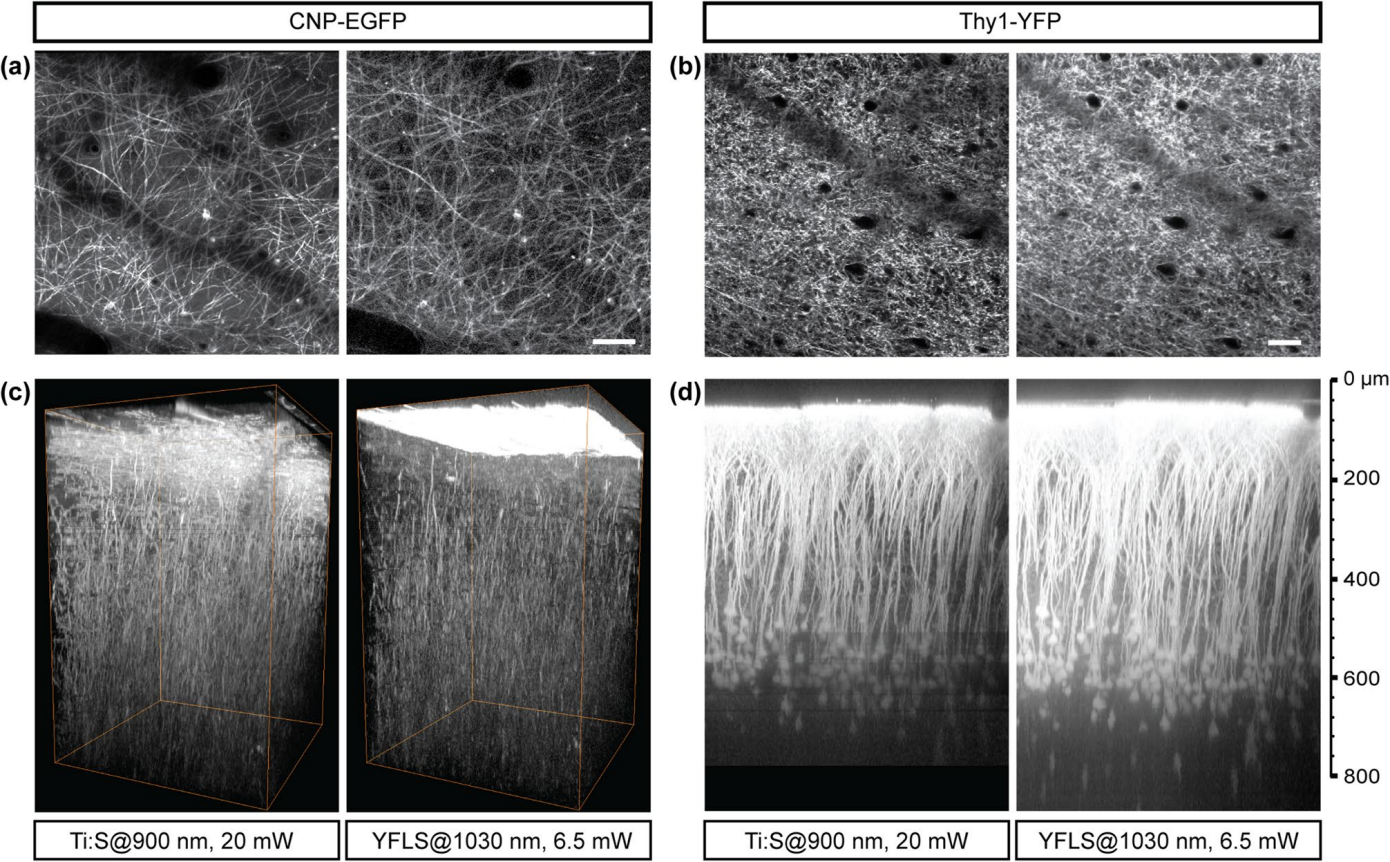
Intravital imaging of transgenic CNP-EGFP and Thy1-YFP brains by the 1030-nm YFLS vs. the 900-nm Ti:Sapphire laser. (**a**,**b**) The superficial cortex on the normalized intensity scales. Scale bars, 50 µm. (**c**) Volumetric rendering of a CNP-EGFP brain with a depth range of 450 µm. (**d**) Thy1-YFP, maximum intensity projection of an axial stack on a logarithmic intensity scale.

Imaging genetically encoded fluorophores has been instrumental for the success of 2PM, allowing specific molecules and cells to be tracked in live animals. For the proof of utility, appropriate fluorescent proteins (FPs) must be identified that are excitable by the YFLS. It is also of great interest whether the vast library of transgenic strains labeled with GFP can be imaged by the YFLS. To this end, we tested two popular FPs, i.e., enhanced GFP (EGFP)^21^ and YFP^22^. Two transgenic strains were employed, namely CNP-EGFP^23^ and Thy1-YFP (H-line)^24^, expressing membrane-anchored EGFP in oligodendrocytes and YFP in neurons, respectively. The brain of an anesthetized mouse was imaged through an optical cranial window. The region of interest was imaged with a setup where the light source alternated between the YFLS at 1030 nm and the Ti:Sapphire laser at 900 nm. The average power was 6.5 mW and 20 mW at the sample, respectively. The two-photon (2P) action cross section (i.e., a product of the 2P absorption cross section and the quantum efficiency of fluorescence) of EGFP has been measured to be approximately 35 GM at 900 nm but negligible at 1030 nm^25,26^. Surprisingly, we found that EGFP was excitable by the YFLS at 1030 nm so that the myelinated fibers as deep as 400 µm in CNP-EGFP could be visualized (Fig. 2a,c). The measured 2P action cross section of a fluorescent protein exhibits substantial variations in the literature^26–29^, or is not available at all for the particular mutant of interest, making it difficult to predict the intravital performance. So we estimated the relative 2P action cross section from the statistics of intravital images, i.e., the standard deviation of the 2PEF signal at the surface

6 $$\sigma \left[{S}_{0}\right]={\eta }_{2}{\Phi }_{0}{\left({P}_{E}\right)}^{2}\sigma \left[N\right]$$

where $N$ is the number of fluorescent molecules. By analyzing the images of the same region, the 2P action cross section of EGFP was determined to be at most ~ 1 GM at 1030 nm, approximately 3% of the value at 900 nm. The estimated value was validated by the relatively equal brightness of the images normalized by ${\eta }_{2}{\Phi }_{0}{\left({P}_{E}\right)}^{2}$ (Fig. 2a). Despite such a low 2P action cross section, bright 2PM images were obtained on account of the high pulse energy of the YFLS. By contrast, YFP could be excited efficiently (Fig. 2b,d). The 2P action cross section of YFP at 1030 nm was approximately 50% of the value at 900 nm (Fig. 2b). As a result, it was anticipated from Eq. (5) that the depth of imaging would increase by 0.34 ${l}_{n}$ compared to that with a 3 times higher average power from the Ti:Sapphire laser. A depth of 2PEF imaging of ~ 860 µm was achieved in a Thy1-YFP brain with a 6.5-mW average power at the sample from the YFLS (Fig. 2d). It was similar to the previous depth obtained with a 200-mW average power at the sample from a 40-MHz YFLS^30^.

### RFP for multi-color intravital 2PM

**Figure 3:**
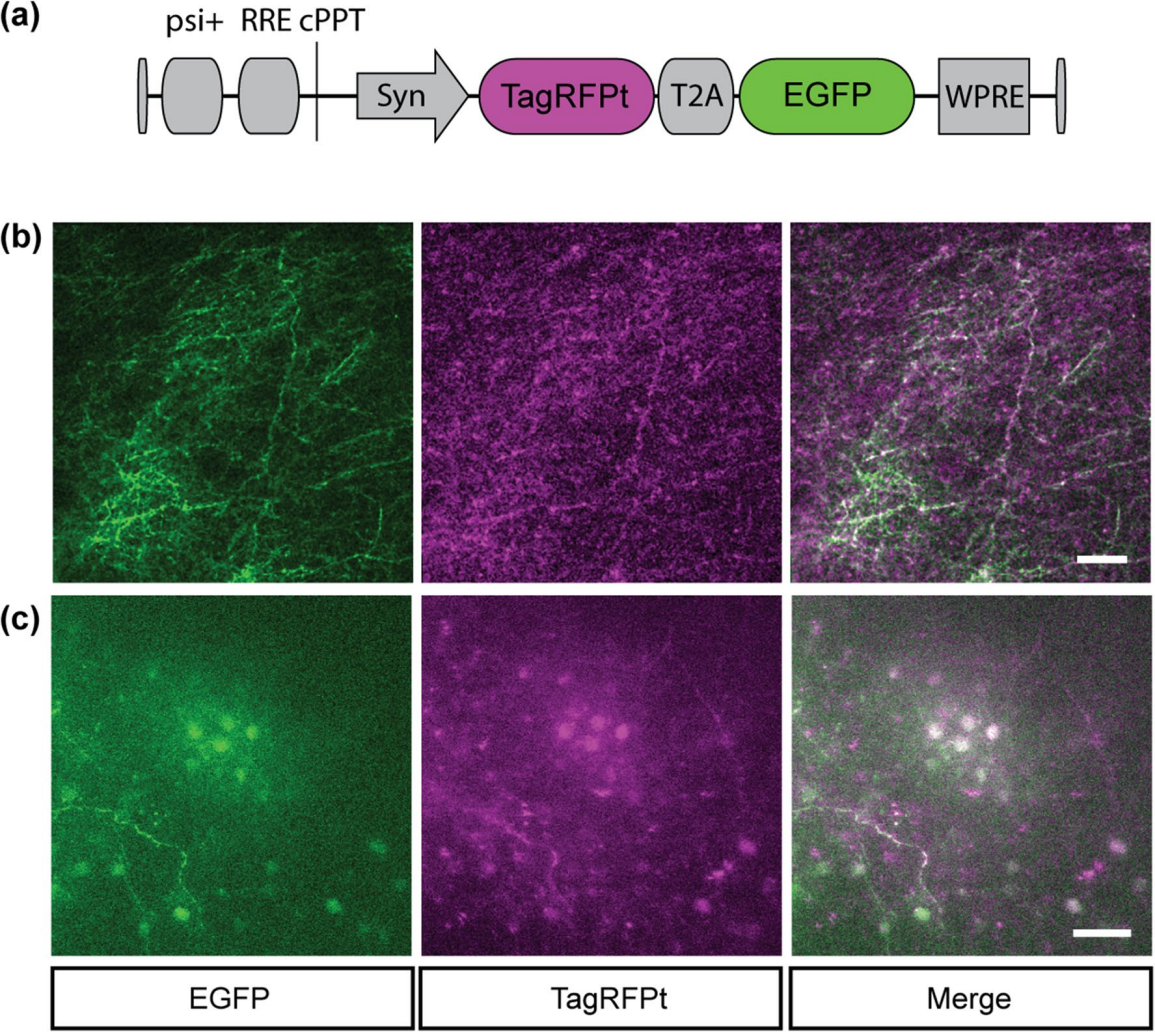
RFP for multi-color intravital 2PM. (**a**) Schematic of pLV-Syn-TagRFPt-T2A-EGFP. (**b**), (**c**) Co-registration of EGFP and TagRFPt, excited by the Ti:Sapphire laser at 900 nm and the YFLS at 1030 nm, respectively. (**b**) The dendrites in the superficial cortex and (**c**) cell bodies at the depth of 300 µm. Scale bars, 50 µm.

The narrow tunability of the YFLS is a drawback, compared to broadband Ti:Sapphire lasers, for multi-color tracking of dynamic interactions between various molecules and cells. We sought to identify additional fluorescent proteins excitable by the new light source. A monomeric RFP TagRFPt, which is an S158T mutant of TagRFP with an enhanced brightness and photostability^31^, was selected because the peak 2P action cross section of TagRFP is at 1030 nm and as high as that of EGFP at 900 nm (~ 35 GM)^25,26^. Furthermore, its variant (mKate2) has been verified for intravital 2PEF imaging of the mouse brain^32^. TagRFPt was expressed in the cerebral cortex via lentiviral transduction. A bicistronic vector was cloned as shown in Fig. 3a: After self-cleaving peptides T2A, EGFP was inserted as a reference fluorophore. The synapsin promoter (Syn) was employed for neuronal expression. After stereotaxic injection of pLV-Syn-TagRFPt-T2A-EGFP, the brain of an anesthetized mouse was imaged through an optical cranial window. EGFP and TagRFPt were co-localized in the dendrites and neuronal cell bodies (Fig. 3b,c). By analyzing a region imaged with the Ti:Sapphire laser at 900 and 1030 nm, it was determined that the relative brightness of TagRFPt at 1030 nm was about one third of that of EGFP at 900 nm. Furthermore, the excitation by the YFLS and the Ti:Sapphire laser at 1030 nm showed that the relative brightness of TagRFPt was reduced by a factor of 3 ~ 4 when excited with high pulse energy. It has been shown previously that the dark state conversion and irreversible photobleaching of TagRFPt depend on the intensity of illumination^33^. The difference we observed between the low and high pulse energy conditions suggested additional photophysical pathways for the latter.

### Photodamages

**Figure 4:**
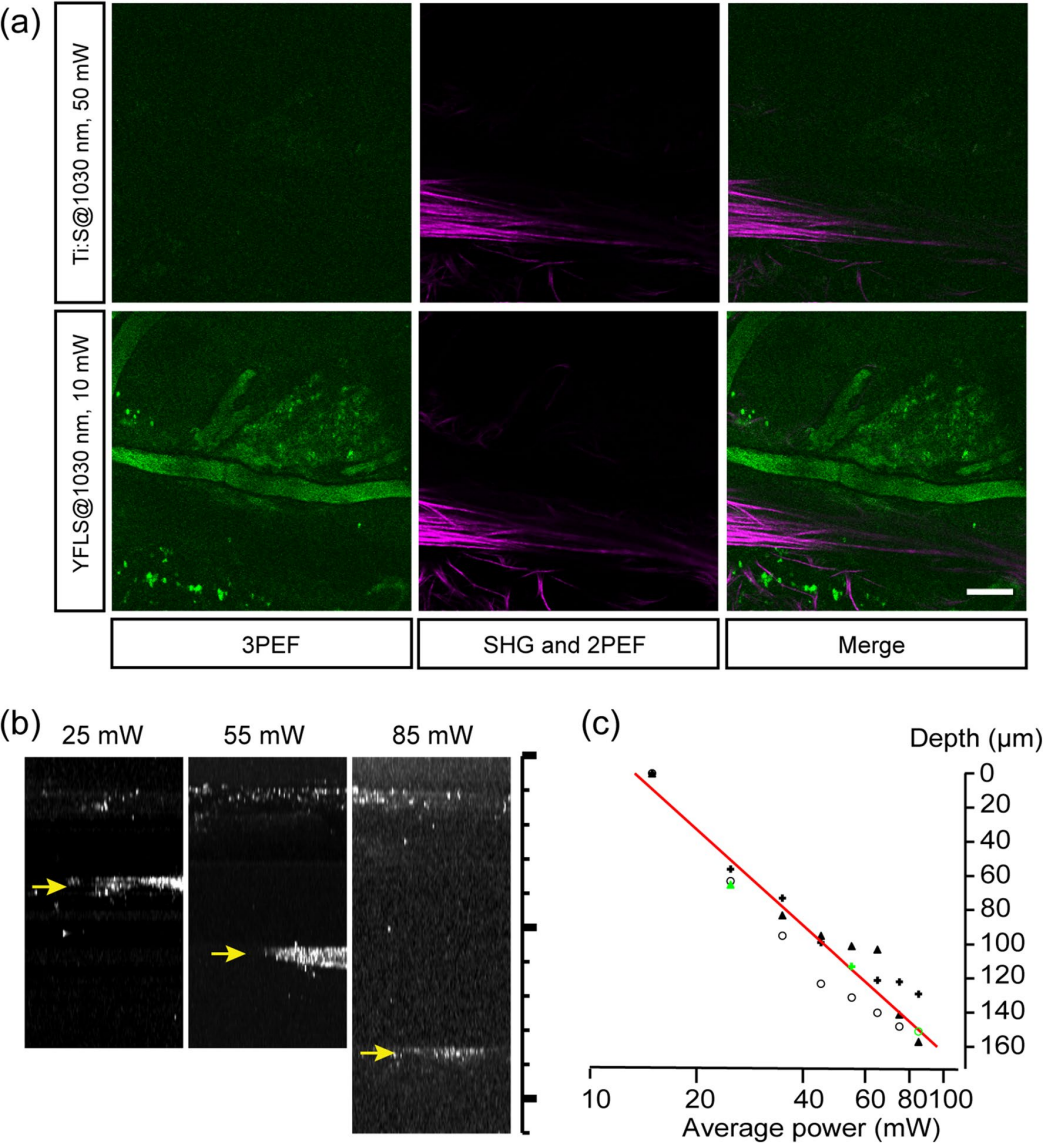
Photodamages in the live brain by high-pulse-energy MPM. (**a**) The superficial cortex imaged by the YFLS and the Ti:Sapphire laser at the same wavelength and 2PE equivalent average power. Scale bar, 50 µm. (**b**) Axial projections showing the depth of photodamage at various average powers (arrows). Scale bar, 220 µm. (**c**) The depth profiles of photodamage (N = 3 mice) and the best fit (red). Green markers indicate the data shown in (**b**).

The depth of high-pulse-energy MPM is limited by various photodamages, including fluorophore-specific photobleaching and tissue-dependent photocytotoxicity. We found that an average power of ~ 10 mW (~ 10-nJ pulse energy) at the sample was safe to the live brain, similar to the previous damage threshold of 25–30 mW for 1-MHz, 300-fs pulses^13^. The corresponding light exposure, i.e., the incident irradiance integrated over time, was approximately 50 J/cm^2^. Substantially above this threshold level, there was an increased propensity of phototoxicity where microbubble-like lesions grew nonlinearly with the exposure time. The damages that occur during 2PM have been suggested to be via high-order nonlinear processes^34,35^. The order of photodamage determines the maximum permissible exposure during high-pulse-energy MPM because the viable peak power can be further restricted. We observed three-photon excited fluorescence (3PEF) from non-labeled live mouse brains similar to the previous report^36^, well below the damage threshold, in the spectral range of common autofluorescence (460–485 nm) shorter than a half of the excitation wavelength. It was specific to high-pulse-energy MPM. When the same region in the superficial brain was imaged by the YFLS and the Ti:Sapphire laser at 1030 nm delivering an identical 2PE equivalent average power (10 mW and 50 mW, respectively), the 3PEF signal was detected only with the YFLS while the 2PEF and SHG signals were comparable (Fig. 4a). Above the damage threshold, light-induced lesions appeared near the positions of intense 3PEF suggesting that phototoxicity was mediated by 3PE. To gain further insights into the order of photodamage, the depth of damage was measured as a function of the average power at the sample. Z-stack images of the live brain were acquired with the 1-MHz YFLS starting from a depth of > 200 µm and moving toward the top of the brain (Fig. 4b). At the onset of photodamage, the average power was abruptly lowered, and the depth was measured. The depth of photodamage can be described by Eq. (5), where the decay length has no contribution from the collection path.

7 $${z}_{damage}={l}_{s}\cdot \mathit{ln}\left[\frac{P}{{P}_{0}}\right]$$

${P}_{0}$ is the threshold average power to cause damages at the surface. The best fit to Eq. (7) yielded the characteristic depth ${l}_{s}$ of 81 µm (Fig. 4c). The threshold average power was approximately 15 mW, which corresponded to the 2PE and 3PE equivalent average power of 75 and 129 mW, respectively, of an 80-MHz source. The observed damage resembled the event at the 3PE rather than 2PE equivalent average power of 80-MHz Ti:Sapphire laser, implicating 3PE as the primary pathway of photodamage. Because of the higher order, the effect of phototoxicity was confined to the superficial layer. It was therefore conceivable that the full depth of high-pulse-energy MPM could be obtained by variable excitation, i.e., gradually increasing the excitation power with depth.

### THG imaging of the mouse brain ex vivo

**Figure 5:**
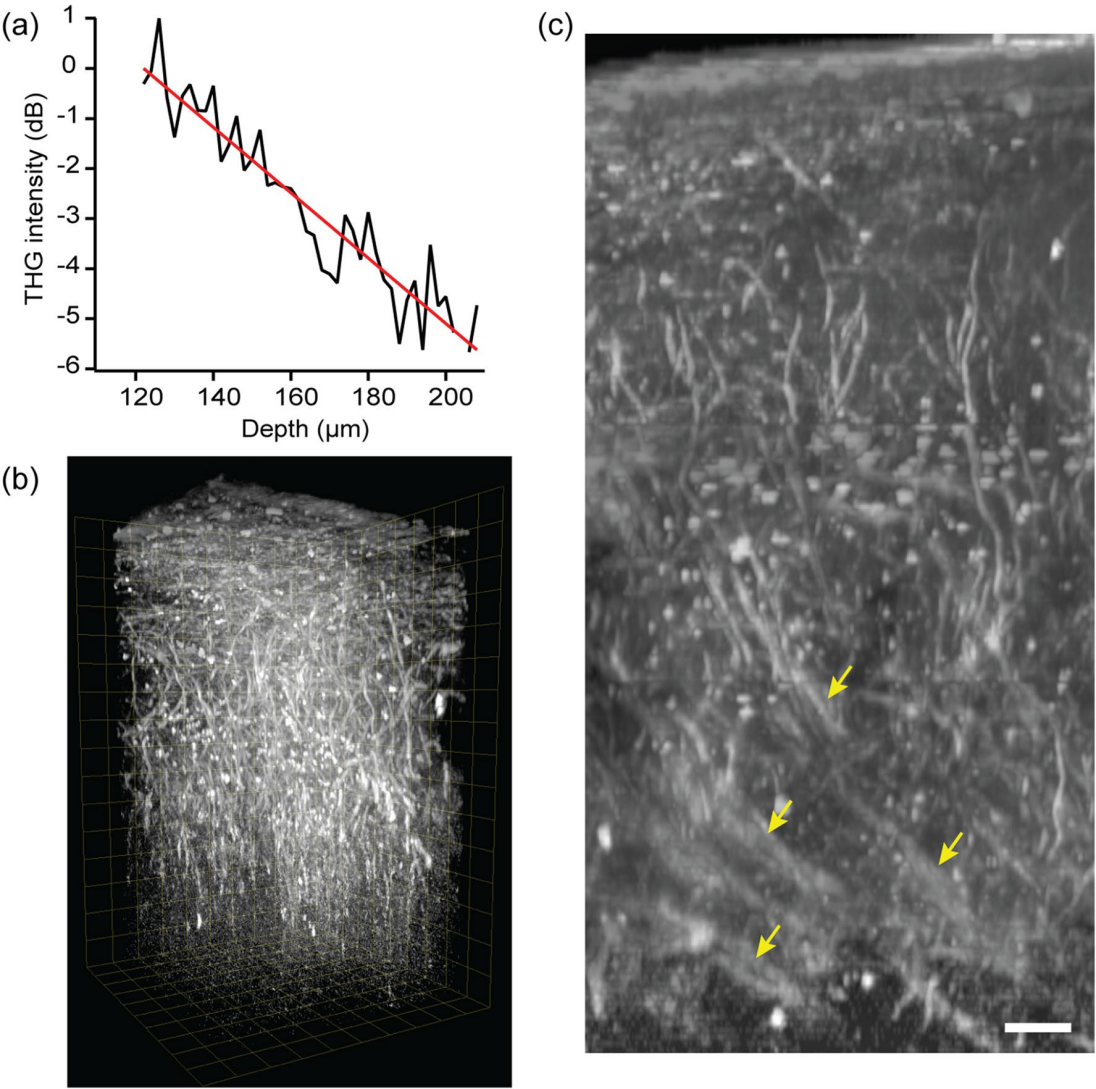
Imaging myelinated axons in the cerebral cortex ex vivo by THG. (**a**) The average profile of THG intensity (N = 7) and the best fit (red). (**b**) Volumetric rendering with a depth range of 400 µm. (**c**) Maximum intensity projection of an axial stack, showing the bundles of radial fibers in layer 4 (arrows). Scale bar, 20 µm.

Intravital THG microscopy has an emerging application, i.e., label-free imaging of the myelinated fibers in the cerebral cortex, for which the short wavelength of a Ti:Sapphire laser is not ideal. Using an optical parametric oscillator (OPO) tuned to 1160 nm and ~ 100 mW of average power at the sample, a depth of ~ 200 µm has been demonstrated^37^. To obtain a deeper range suitable for visualizing myelination by oligodendrocytes across the cortical layers, we performed high-pulse-energy THG imaging. Equation (5) predicts that the depth of THG imaging of ~ 500 µm is achievable with an average power of 50 mW from the 1-MHz YFLS, i.e., an additional depth of $4.4{l}_{n}$ compared to that achieved by the 100-mW, 80-MHz OPO. However, the decay length ${l}_{n}$ could be reduced due to the stronger Rayleigh scattering and absorption of the ultraviolet THG emission (Eq. (3)). We examined the decay length of high-pulse-energy THG imaging using fixed brains. The average THG intensity of single myelinated axons was analyzed (Fig. 5a). The best fit to Eq. (2) yielded a decay length of 67 µm, which was a little shorter than the value obtained with an OPO (Table 2). Next, we tested the maximum depth of THG imaging. A variable excitation was employed in which the average power started from 50 mW and gradually decreased as the image plane moved toward the top of the brain. Myelinated fibers up to 400 µm deep were visualized within the intact brain (Fig. 5b and Suppl. Movie S1), which was approximately 2 times deeper than the result from an OPO despite using only half the average power. Consequently, the bundles of myelinated fibers in layer 4 could be visualized in the whole intact brain (Fig. 5c).

**Table 2:** The decay lengths of MPM imaging of the rodent brains.

	Decay length (µm)	Wavelength (nm)	References
2PEF	65	775	^38^
	~ 100	800	^19^
	80–100	800–850	^39^
	120–220	830	^40^
	100	~ 860	^41^
	80	850, 920	^59^
	95	925	^12^
	170	940	^14^
	126	1110	^59^
	143	1280	^38^
THG	67	1030	This work
	65–84	1160	^37^
3PEF	122	1675	^42^
Photodamage^a^	81	1030	This work

^a^The characteristic length at the excitation wavelength.

## Discussion

We have demonstrated high-pulse-energy MPM imaging of the murine brain. Increased MPM signal and enhanced depth were obtained with a pulse energy > 10 times higher than achieved by the standard setup with a Ti:Sapphire laser. The YFLS was evaluated against Ti:Sapphire lasers by means of the fold change in the equivalent average power. A factor of 5 and 8.6 was demonstrated for 2PE and 3PE, respectively. The depth of 860 µm was obtained using 6.5 mW of average power for imaging the Thy1-YFP brain in vivo, and 400 µm using 50 mW for THG imaging of cortical myelin ex vivo. Further improvement by a factor of 10 and 21 for 2PE and 3PE, respectively, seem feasible by lowering the repetition rate to 250 kHz at which the number of pulses per pixel is ~ 1 for a frame of 512 × 512 pixels and a rate of 1 Hz, provided that the superficial photodamages are properly managed, e.g., by means of modulating the average power gradually with depth. It could then visualize the entire gray matter of the cerebral cortex. High-pulse-energy MPM therefore represents an orthogonal approach to wavefront compensation^43,44^. We measured the decay lengths, ${l}_{n}$, of the high-pulse-energy MPM of the mouse brain which were within the range of values in the literature (Table 2).

High-pulse-energy MPM presents opportunities as well as challenges. New regimes of light-molecule interactions could be utilized for microscopic imaging, e.g., the 2PE of fluorescent molecules with 2P action cross sections as low as < 1 GM. By contrast, it also elicits nonlinear photodamages which are relatively insignificant under the ordinary 2PM condition. While the use of low average powers is advantageous for reducing the effect of 1PE-induced photodamages, such as heating^45^, we found that phototoxicity was mediated primarily by higher order (3PE) processes. The safety of high-pulse-energy MPM must be characterized for long-term time-lapse imaging and the possible mechanisms of phototoxicity need to be further elucidated, e.g., the activation of flavin-containing oxidases^46^ and the generation of reactive oxygen species^47^. Various techniques could help mitigate phototoxicities in high-pulse-energy MPM^48,49^. Another challenge for high-pulse-energy MPM concerns the speed of imaging^12^. The required number of pulses per pixel imposes a limit less favorable than for the 80-MHz Ti:Sapphire lasers. The maximum line-scan rate of ~ 4 kHz, or the maximum frame rate of ~ 15 Hz (1 pulse per pixel and 256 × 256 pixels per frame), is still adequate for resolving the Ca^2+^ transients (~ 100 ms) by line-scanning but not by frame-scanning.

A variety of RFPs have been developed^31,50–54^, including Ca^2+^ indicators^55,56^, to improve the depth and safety of intravital imaging. We found that TagRFPt was modestly appropriate for intravital 2PM imaging, although the brightness was compromised under the illumination with high pulse energy. The excitation-driven photophysics of TagRFPt, similar to YFP^57^, may underlie the observed reduction in the brightness. It is conceivable that other variants of TagRFP (e.g., R67K S158T)^33^, or new RFPs specifically evolved for high-pulse-energy MPM, could improve the utility of the YFLS.

The YFLS has gained much attention for 2PE due to the wavelength filling a spectral gap between the Ti:Sapphire laser and the OPO^58,59^. Taken together, our results demonstrate its potential as a stand-alone light source for high-pulse-energy MPM. Comparable MPM signals could be obtained at a low average power; typically, less than 100 mW was necessary directly out of the laser system except for THG imaging. The reduced average power is a great advantage for designing a simpler, affordable source for MPM.

## Methods

### Animals

All mice were obtained from Jackson Lab, including C57BL/6, Thy1-YFP (#003782) and CNP-EGFP (#026105) mice. All experimental protocols were approved by the Hunter College Institutional Animal Care and Use Committee (IACUC). All methods were carried out in accordance with relevant guidelines and regulations. The study was carried out in compliance with the Animal Research: Reporting of In Vivo Experiments (ARRIVE) guidelines.

### Cloning of LV transgene

The third generation LV vector was used (2 μg, Addgene #14883) for cloning pLV-Syn-TagRFPt-T2A-EGFP. The vector was digested by restriction enzymes and purified by gel electrophoresis. The inserts of TagRFPt-t2A and EGFP were amplified by PCR using Pfx DNA polymerase (Thermo Fisher Scientific). The inserts and the vector were ligated using HiFi DNA Assembly (New England Biolabs). Bacterial cells were transformed. 10 colonies were selected and grown in LB media overnight. The result of ligation was verified by restriction digest and Sanger sequencing.

### Production and concentration of LV

High titer LVs were produced as described previously^32,60^. Briefly, approximately ~ 300 μg of transfer vector was obtained by growing bacteria in 500-mL LB media overnight and maxi-prep. 293 T cells were seeded in twelve 15-cm dish approximately 8 h prior to transfection. The medium was changed 2 h prior to transfection. The mixture of plasmids (transfer vector, pMDLg/pRRE pREV, and pVSV-G) was prepared for transfection. Then DNA-CaPO_4_ precipitate was formed by adding CaCl2, double distilled H2O, and 2 × HBSS. After incubating for 12–16 h, the precipitate was removed, and the media was changed. After incubation overnight, the supernatant was collected for the first harvest. Fresh 15-mL media was added and incubated overnight. The supernatant was collected for the second harvest. The supernatants were pooled and cleaned up with 0.45-μm filter. At this point the titer was > 10^6^ viral particles/mL. After concentration by ultracentrifuge the titer increased ~ 150-fold to > 10^8^ viral particles/mL.

### Stereotaxic injection

LV was injected using a stereotaxic frame as described previously^32,61^. A mouse was anesthetized with 1.5% isoflurane at 0.5 L/min oxygen delivery. The mouse was placed in a stereotaxic frame and the head was firmly secured with ear bars. A small flap of skin was removed with a scalpel from the dorsal skull of the visual cortex. A hole of 300 μm in diameter was drilled above the target area. The tip of a 32-gauge needle was lowered to a desired depth below the pia mater. Then a 1-µL solution of LV particles was injected slowly over 10 min. After 2 min, the needle was withdrawn slowly, and the wound was closed by clipping the skin. The animal received post-operative care.

### Installing the cranial optical window

An optical cranial window was placed as described previously^32,62^. A mouse was anaesthetized with 1.5% isoflurane, the head immobilized, and placed on a heating blanket. An eye ointment was applied to prevent drying. The mouse was placed in a stereotaxic frame and the head was firmly secured with ear bars. The area of operation was sterilized with betadine and then 70% ethanol. The skin over the head was cut using scissors. The exposed area was scraped with a scalpel for better adhesion. A 2-mm guide circle was drawn with a pencil which was subsequently drilled until a thin layer of skull was left. A drop of phosphate-buffered saline (PBS) was put on the area and the thinned skull was removed using the tip of a thin forceps. After the dura was dry, a sterile 3-mm glass coverslip was placed on top of the dura mater and glue was applied around the coverslip. Dental acrylic mix was applied around the edges of the cover slip to cover the entire skull surface. A titanium head bar or razor blade was placed on the acrylic resin and allowed 10 min to harden in order to reduce motion artifacts.

### The retinal flatmount

The retinal flatmounts were prepared as described previously^20^. The mouse was euthanized, and the eye was enucleated. The retina was peeled off and radial cuts were made. The retina was placed on a glass-bottom dish and immersed into oxygenated PBS solution for imaging.

### Intravital MPM imaging

A standard setup was used as described previously^32,63^. Short pulses from the YFLS (constructed at Clark-MXR) and the Ti:Sapphire laser (Chameleon Ultra, Coherent) were used. The elliptical polarization was obtained with half- and quarter-waveplates. For the YFLS path, a telescope of two concave mirrors was used to magnify the beam. The magnification was chosen to properly fill the entrance pupil of the objective lens. For THG imaging, an objective lens was used which had high transmission at both the excitation and emission wavelengths and a PMT was used that had an adequate sensitivity at 343 nm. The excitation beam was focused with a water-dipping objective lens (Olympus LUMPLFLN40XW 0.8NA for THG and Leica HC FLUOTAR L 25 × 0.95NA for all others). That the excitation beam was free from aberrations was confirmed by measuring the point spread function (PSF) with sub-resolution fluorescent beads. The average power was measured with two independent sensors. The backward-scattered signal from the brain was collected with the same objective lens and detected with photomultiplier tubes (PMT; Hamamatsu H10722-210 for THG and Hamamatsu H10770PA-40 for all others). The signal was collected after narrow bandpass filters by non-descanned epidetection. The center/bandwidth of filters were: 340/22 (THG), 420/40 (photodamage), 473/24 (3PEF), 525/25 (SHG), 525/50 and 525/70 (EGFP and YFP), and 605/70 (TagRFPt). The pixel dwell time was ∼4 μs. Typically 1–5 frames were acquired at the frame rate of ∼1 Hz. After imaging, the animal was euthanized by CO_2_ inhalation.

### Image processing

Images were processed with ImageJ^64^. Myelinated fibers were traced by single-particle tracking^65,66^. For THG images acquired with variable excitation, the contrast of z-stack was adjusted prior to 3D visualization^67^. Volumetric rendering was created using Amira (Thermo Scientific).

## Supplementary Information

Supplementary Information.

Supplementary Video.

## Data Availability

The datasets generated during and/or analyzed during the current study are available from the corresponding author on reasonable request.
