# Author Correction: High-pulse-energy multiphoton imaging of neurons and oligodendrocytes in deep murine brain with a fiber laser

**DOI:** 10.1038/s41598-021-95097-1

**Published:** 2021-08-23

**Authors:** Michael J. Redlich, Brad Prall, Edesly Canto-Said, Yevgeniy Busarov, Lilit Shirinyan-Tuka, Arafat Meah, Hyungsik Lim

**Affiliations:** 1 Department of Physics and Astronomy, Hunter College, New York, NY 10065 USA; 2 Department of Physics, The Graduate Center of the City University of New York, New York, NY 10016 USA; 3 Clark-MXR, Inc., 7300 W. Huron River Drive, Dexter, MI 48130 USA

Correction to: *Scientific Reports* 10.1038/s41598-021-86924-6, published online 12 April 2021

The original version of this Article contained extensive reference errors, where the references given in Table 2 were incorrect.

The authors omitted the below papers, which are now listed as References 38–42.

Kobat, D. *et al.* Deep tissue multiphoton microscopy using longer wavelength excitation. *Opt. Exp.* **17**, 13354–13364, https://doi.org/10.1364/oe.17.013354 (2009).

Kleinfeld, D. & Denk, W. in *Imaging Neurons—A Laboratory Manual* Vol. 23 (eds R. Yuste, F. Lanni, & A. Konnerth) 23.21–23.15 (CSHL Press, 2000).

Kleinfeld, D., Mitra, P. P., Helmchen, F. & Denk, W. Fluctuations and stimulus-induced changes in blood flow observed in individual capillaries in layers 2 through 4 of rat neocortex. *Proc. Natl. Acad. Sci. U.S.A.* **95**, 15741–15746 (1998).

Denk, W. *et al.* Anatomical and functional imaging of neurons using two-photon laser-scanning microscopy. *J. Neurosci. Methods* **54**, 151–162 (1994).

Horton, N. G. et al*.* In vivo three-photon microscopy of subcortical structures within an intact mouse brain. *Nat. Photonics* **7**, 205–209, 10.1038/nphoton.2012.336 (2013).

Consequently, References 43–67 were incorrectly listed as References 38–62.

The original Table 2 and accompanying legend appear below.

**Table 2:** The decay lengths of MPM imaging of the rodent brains. ^a^The characteristic length at the excitation wavelength.

	The decay length	Wavelength	Reference
2PEF	65 µm	775 nm	^38^
	~ 100 µm	800 nm	^19^
	80–100 µm	800–850 nm	^39^
	120–220 µm	830 nm	^40^
	100 µm	~ 860 nm	^41^
	80 µm	850, 920 nm	^59^
	95 µm	925 nm	^12^
	170 µm	940 nm	^14^
	126 µm	1110 nm	^59^
	143 µm	1280 nm	^38^
THG	67 µm	1030 nm	This work
	65–84 µm	1160 nm	^37^
3PEF	56 µm	1675 nm	^42^
Photodamage^a^	81 µm	1030 nm	This work

The original Article has been corrected.

