# Supplementary material for: High-pulse-energy multiphoton imaging of neurons and oligodendrocytes in deep murine brain with a fiber laser
Source: Sci Rep. 2021 Apr 12;11:7950. doi: 10.1038/s41598-021-86924-6 (PMC8041775; doi:10.1038/s41598-021-86924-6)

Title: High-pulse-energy multiphoton imaging of neurons and oligodendrocytes in deep murine brain with a fiber laser

Authors: Michael J. Redlich[1,2], Brad Prall[3], Edesly Canto-Said[3], Yevgeniy Busarov[1], Lilit Shirinyan-Tuka[1], Arafat Meah[1], and Hyungsik Lim[1,2*]

[1]Department of Physics and Astronomy, Hunter College New York, NY 10065

[2]Department of Physics, The Graduate Center of the City University of New York, New York, NY 10016

[3]Clark-MXR, Inc., 7300 W. Huron River Drive, Dexter, MI 48130

*Correspondence should be addressed to Hyungsik Lim, hyungsik.lim@hunter.cuny.edu

Supplementary Movie S1. Volumetric rendering of myelinated axons in the mouse cortex ex vivo visualized by THG. The depth range is 400 µm.

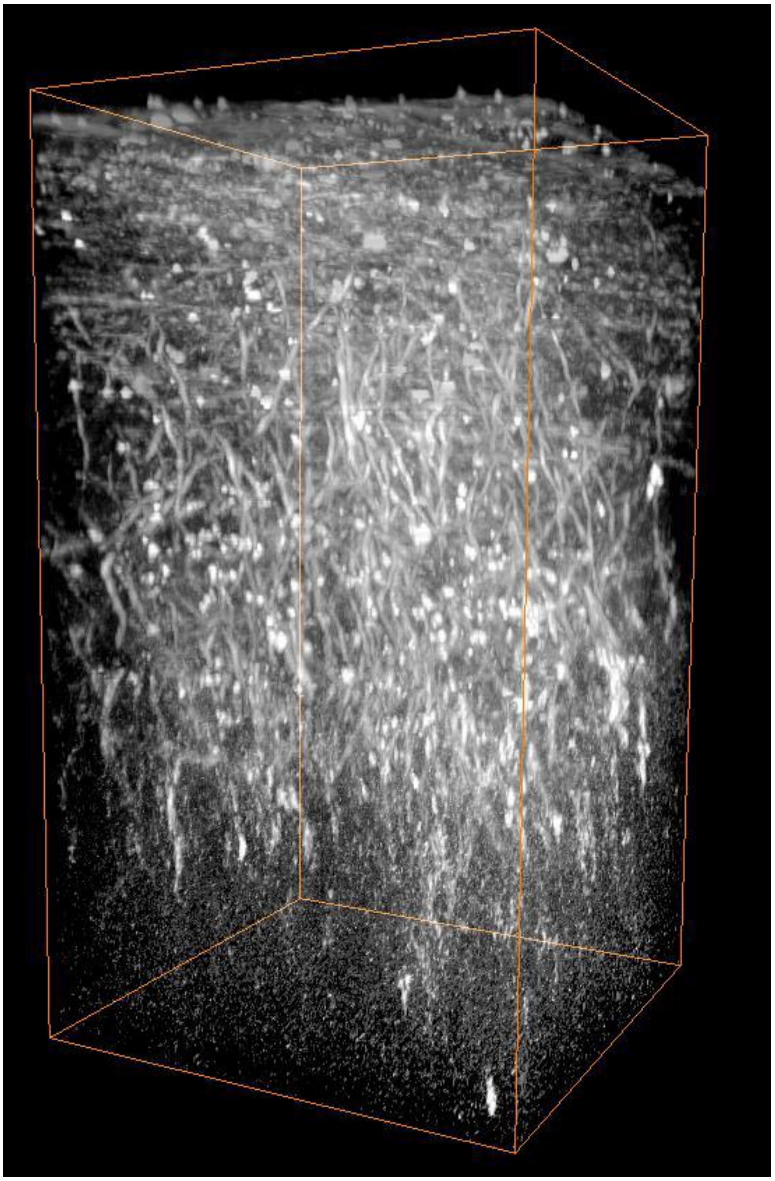

Supplement: Supplementary file 1 — Supplementary Information. [file 41598_2021_86924_MOESM1_ESM.pdf]
